# Ankle fractures of the geriatric patient: a narrative review

**DOI:** 10.1530/EOR-22-0082

**Published:** 2023-01-27

**Authors:** Patrick Ziegler, Christian Bahrs, Christian Konrads, Philipp Hemmann, Marc-Daniel Ahrend

**Affiliations:** 1 BG Klinik Tübingen, Department of Traumatology and Reconstructive Surgery, Eberhard Karls University of Tübingen, Tübingen, Germany; 2 Department of Orthopaedics and Trauma Surgery, Klinik Gut, St. Moritz, Switzerland; 3 Schön Klinik Neustadt, Neustadt in Holstein, Germany; 4 Department of Orthopaedic Surgery, University of Tübingen, Tübingen, Germany; 5 AO Research Institute Davos, Davos Switzerland

**Keywords:** geriatric fracture, ankle fracture, osteoporosis, fragility fracture, fibular nail

## Abstract

- The present narrative review provides a summary of current concepts for the treatment of ankle fractures in elderly patients.
- Despite a high complication rate, open reduction and internal fixation is the gold standard for operative care. However, individual patient-based treatment decision considering the soft-tissue status, the fracture pattern, as well as the patient’s mobility and comorbidities is mandatory to achieve sufficient patient outcomes.
- Due to high complication rates after surgery in the past, techniques such as fibular nails or minimal invasive techniques should be considered.

## Introduction

Ankle fractures represent one of the most common injuries in elderly patients combined with a major health care burden as the elderly population is expected to more than double by the year 2050 (1, 2, 3, 4). The overall 1-year mortality of elderly patients (>65 years of age) with an ankle fracture is 12% (5).

Open reduction and internal fixation (ORIF) normally leads to fair postoperative results in young patients with an ankle fracture and is therefore widely accepted. However, optimal treatment for geriatric fractures remains controversial. Geriatric patients are particularly at risk of poor outcomes following ankle fractures due to frequent multimorbidity, poor peripheral blood supply and osteoporosis (6, 7). Complications involve loose intraoperative fixation related to reduced screw purchase and soft tissue defects with wound healing deficits or malunion (8).

Based on the large heterogeneity of geriatric patients with comorbidities, different fracture patterns and pre-traumatic activity level, individual patient-based treatment decision is mandatory to reduce complication rates and to achieve sufficient patient outcomes. The present review provides a summary of current concepts for the treatment of ankle fractures in the elderly.

## Diagnostics

During the anamnesis, besides the circumstances of the accident and cause of the fall, it is particularly important to ask geriatric patients for comorbidities, like diabetes mellitus, arterial disease, dementia, osteoporosis, past events of falls and fractures, neurological deficits, medication, degree of mobility before the accident and social environment. These comorbidities have high impact on the treatment decision and treatment complications (8). Especially in geriatric patients, during the clinical examination, pulses have to be palpated, and Doppler and duplex sonography of peripheral arteries is recommended (9). In case the pulses are not palpable, the Doppler sonography or the ankle-brachial index is pathologic, and a CT angiography is recommended (9). If the CT angiography reveals relevant vascular stenosis which negatively affects limb perfusion, a percutaneous transluminal angioplasty either stand-alone or combined with stent implantation should be performed prior to the surgical fracture fixation to improve limb perfusion and reduce the likelihood of postoperative complications (9).

The clinical examination of the ankle usually shows a painful swelling of the affected ankle area. Similar to ankle fractures in younger patients, the entire fibula has to be examined to avoid missing high fibula fractures. Further assessment of soft tissue conditions with closed or open tissue damage is important. Besides the fracture pattern and comorbidities, the soft tissue conditions determine further therapy regimes (10). A possible dislocation of the ankle joint should be directly reduced under appropriate analgesia. Otherwise, there is a high risk of trophic disorders around the ankle following skin and soft tissue necrosis. Imaging diagnostics should be done immediately after reduction and retention.

Rest, application of ice, compression and elevation (RICE scheme) of the limb is generally recommended to reduce further swelling. However, the evidence level for the optimal initial emergency treatment of ankle fractures is low (11). The RICE recommendations have not been rigorously investigated (12) and are mainly examined on healthy subjects or animals (13). Furthermore, care has to be taken in patients with comorbidities such as dementia, peripheral nerve disorder or peripheral arterial disease. Hypothermic injuries can further compromise the soft tissue (11). In doubt of patient compliance with cooling instructions, cooling and compression should be avoided. An alternative is the use of arteriovenous impulse systems. These systems reduce the post-traumatic swelling after ankle fractures and decrease the time from trauma to definitive surgery and reduce the risk of wound healing complications (14).

The primary imaging technique for acute ankle injuries is a conventional X-ray of the affected lower leg (15). For most acute injuries, native X-rays are performed of the upper ankle joint in two planes, anteroposterior and lateral, on the unloaded foot (16). For standard X-rays in an anteroposterior view, the lower leg must be internally rotated by 20° so that the correct position of the ankle can be assessed (‘mortise view’) (17). If a high fracture of the fibula (Maisonneuve fracture) is suspected, such as additional pain in the proximal lower leg, dehiscence of the ankle joint or fracture of the medial malleolus without a visible distal fibular fracture, a complete image of the affected lower leg should always be performed.

Complex fracture patterns, such as multifragment involvement of the lateral malleolus, additional fracture of the medial malleolus, involvement of the dorsal tibial facet or bony avulsion of the syndesmosis are common in ankle fractures of the elderly due to osteoporotic bone density (18). Especially, the sensitivity of multifragment distal fibular fractures on conventional radiographs is low (18). In complex fractures with involvement of the joint surface or the posterior malleolus, the use of a CT scan provides better preoperative planning. Therefore, advanced imaging using a CT scan to fully assess the fracture extent is recommended (19, 20).

## Classification

Ankle fractures in elderly patients can be classified according to a young patient’s cohort. In everyday clinical practice, distal fibular fractures are often classified by the Weber classification (21). This classification is based on the height of the fibular fracture in relation to the anterior syndesmosis and distinguishes three types of fracture levels, fibular lesion distal to the syndesmosis, fibular lesion at the level of the syndesmosis and fibular lesion proximal to the syndesmosis. Accordingly, the classification involves type A, B and C fractures. The syndesmosis is always injured in Weber C fractures, might be injured in type B fractures and is normally uninjured in type A fractures.

Another classification for ankle fractures is the Lauge-Hansen classification (22). It is based on the circumstances of the accident and the resulting forces applying to the foot. There are four different fracture types: fracture in supination/adduction, supination/eversion, pronation/abduction and pronation/eversion. The classification attempts to correlate injury mechanisms to specific fracture patterns. However, the reliability is limited, and the injury mechanism is often speculated by the patient. Nevertheless, the classification underlines the importance of recognizing ankle ligament injuries. Especially for geriatric patients, the classification can be used for closed fracture reduction using the reversed injury mechanisms to avoid unsuccessful or poor fracture reduction with soft tissue damage (23).

Ankle fractures can be subdivided into unimalleolar, bimalleolar or trimalleolar fractures. Trimalleolar fractures with the involvement of a posterior malleolar fragment account for approximately 7% of all ankle fractures (24). In Weber B and C fractures as well as fracture dislocations of the ankle, the posterior tibial rim is involved in approximately 46% of cases (25). The incidence of these posterior malleolar fragments increases especially for elderly (>65 years of age) women with correlated poorer outcomes (26). The shape and the volume of the posterior malleolar fracture differ due to the injury mechanism (27). A frequently used classification for posterior malleolar fragment fractures was described by Haraguchi *et al.* (28). Three main fracture patterns were described on axial CT scans. Type I is a posterolateral-oblique fracture which involves a wedge-shaped fragment at the posterolateral corner of the tibial plafond. Type II is characterized by a transverse fracture line extending from the fibular notch of the tibia to the medial malleolus. In this fracture type, there may be more than one fragment. Type III fractures are defined by one or more small shell-shaped fragments of the posterior tibial lip. These fragments may be too small to be fixated.

Another more comprehensive classification of posterior malleolar fractures was described by Bartoniček *et al.* (20). The classification is based on CT reconstructions. It takes the size, shape and location of the fragment, the stability of the tibiotalar joint and the integrity of the fibular notch into account. Five subtypes are characterized: type 1: extraincisural with an intact fibular notch (corresponding to Haraguchi type III), type 2: posterolateral fragment extending into the fibular notch, type 3: posteromedial two-part fragment involving the medial malleolus (corresponding to Haraguchi type II), type 4: large posterolateral triangular fragment and type 5: irregular, osteoporotic fragments. The classification of Haraguchi *et al.* (28) and Bartoniček *et al.* (20) is useful for the indication for surgery and the choice of incision.

The AO classification of ankle fractures is a further development of the Weber classification and enables the most precise description of the injury pattern. It should therefore be mainly used for both young and elderly patients.

## Treatment options

The primary goal in treating ankle fractures in elderly patients is to restore health-related quality of life as soon as possible as well as to avoid treatment complications and immobility. This can be reached by obtaining osseous union and restoring a stable ankle joint resulting in a pain-free ankle so that the patient can early return to his preinjury activity level. The prevention of post-traumatic osteoarthritis of the ankle joint is less important compared to the younger population.

Suboptimal bone density with poor peripheral blood supply and compromised soft tissue may restrict internal fixation opportunities and may require other options like external fixator stabilization, minimal invasive techniques or prolonged splinting and casting, although this could lead to less stability or soft-tissue damage (29). Most of the complications during the treatment of ankle fractures are related to soft tissue problems (30). Soft tissue conditions should be classified following the Tscherne classification (31).

## Conservative treatment

Conservative treatment of ankle fractures often leads to low satisfaction (32). Furthermore, several studies on large cohorts of elderly patients reported that conservative treatment of ankle fractures was associated with increased mortality rates (4, 33). However, conservative treatment might be indicated for elderly patients with low functional demands (33), long-standing neuropathy with comorbidities or restricting walking distance. These patients will likely tolerate some anatomic displacement (19, 34). Therefore, it is very important to have information about the medical and social background of the patient and consider this information for the appropriate individualized treatment plan (19). Osseous and ligamentous syndesmotic instabilities must be excluded prior to conservative treatment such as posterior malleolar fractures. Isolated fractures of the medial or lateral malleolus (Weber A and B) can be treated conservatively if they are stable and not dislocated (<2 mm) (35). Successful closed reduction before cast immobilization can enhance treatment outcomes (36). Conservative treatment of stable medial or lateral malleolus fractures (Weber B/AO type A1 or B1) requires good compliance and includes immobilization in a lower leg cast for at least 6 weeks combined with partial weight-bearing of 20 kg on crutches or a wheeled walker. Some surgeons suggest early full-weight-bearing for stable fractures after the swelling subsided as higher quality of life and functionality can be achieved (37, 38, 39).

During immobilization, regular soft tissue examinations must be performed in order to avoid pressure marks or even worse soft tissue complications (40). Follow-up radiographs are performed after 1 week to detect possible secondary dislocations and after 6 weeks prior to cast removal (41). However, increments between radiographs can be adjusted dependent on the fracture type and the comorbidities of the patient. However, routine radiographs seldom alter the treatment strategy for ankle fractures (42).

## Surgical treatment

All unstable fractures of the medial and lateral malleolus and fractures of the posterior malleolar fragment should be treated surgically as insufficient reduction and/or incongruence of the ankle joint correlate with poor functional results (43).

Timing of definitive surgery is dependent on the soft tissue. Surgical definitive treatment should be performed when the swelling is subsided. Daily judgment of the soft tissue status is mandatory as swelling reduction show a high individual variability (44). Aigner *et al.* (44) analyzed 237 geriatric ankle fractures treated with ORIF retrospectively. Time from trauma to definitive surgery lasted 6.7 ± 4.2 days ranging from 0 to 35 days. Time from trauma to surgery was not associated with higher complication rates if the swelling is carefully evaluated (44, 45). However, the amount of swelling is correlated with wound complications after operative treatment of ankle fractures (45). If the soft tissue condition permits surgical intervention, early surgery should be attempted to reduce the duration of hospital stay (46).

The use of tourniquets, even in geriatric patients with uninjured soft tissue conditions, should be performed carefully and, if possible, avoided, because it is associated with the development of nerve palsies, ischemic muscle damage and wound complications (34). Careful soft tissue management cannot reduce the primary extent of soft tissue injury, but the extent of secondary damage can be decreased. Surgeons should consider the use of low-profile forceps to minimize compressive forces on tissue and aim for tension-free wound closure with the Allgöwer-Donati technique. In a pig model, it was shown that more cutaneous blood flow can be maintained with this suture technique compared to simple suture patterns, vertical or horizontal mattress configurations (34, 47, 48).

## Open reduction and internal fixation

**Figure 1:**
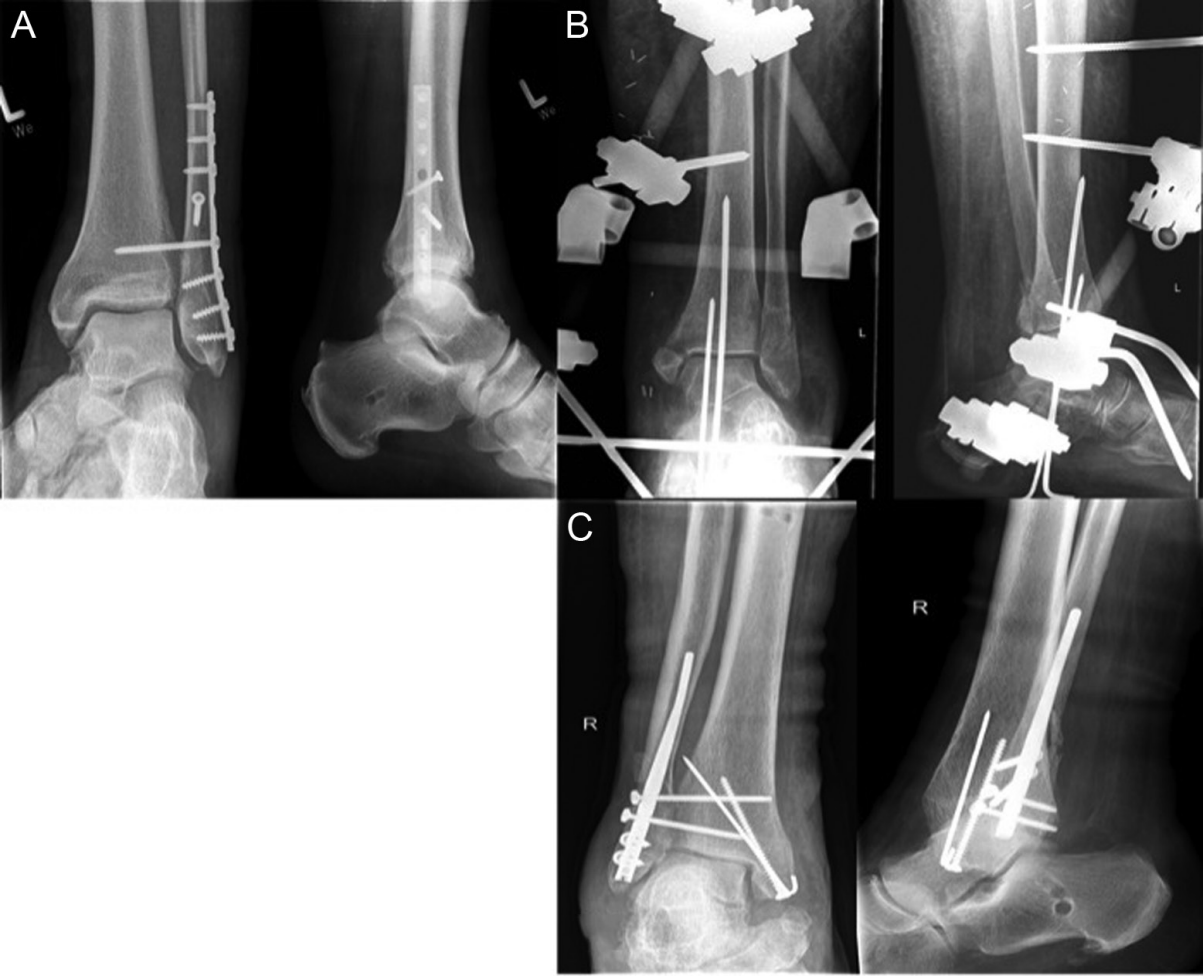
(A) Suprasyndesmotic fibula fracture after open reduction and internal fixation and stabilization with a positioning screw; (B) fibula fracture with a medial malleolar fracture and fracture of the posterolateral rim (Volkmann’s fragment) after closed reduction and stabilization with an external fixator and retrograde K-wires; (C) transsyndesmotic fibula fracture with a medial malleolar fracture after closed reduction of the distal fibula and stabilization with a retrograde fibular nail and open reduction of the medial fracture and stabilization with a compression screw and a K-wire.

The aim of surgical treatment (Fig. 1) of the fractured distal fibula is the correct restoration of the fibular length, torsion and axis as well as stable retention. Normally, distal fibular fractures are fixed with a lag screw and a neutralization plate after open anatomic reduction (49). In multifragment fibular fractures or osteoporotic bone, distal fibular locking plates show biomechanical advantages over traditional plating and should be considered for geriatric patients with poor bone stock as complication and revision rates can be reduced (50, 51, 52).

There are different possible approaches depending on the fracture pattern. In patients with osteoporotic bone, bone avulsions of the tibiofibular syndesmosis are more likely than a ligamentous injury of the syndesmosis (53). Anatomical reduction and fixation of the posterior fragment results in a stable syndesmosis (54). Based on the Bartoniček classification (20), the treatment and the approach to the posterior malleolar fragment can be chosen (55, 56). Type I and undisplaced type II and III fractures can be treated conservatively. Type II and type III fractures with displacement, intercalary fragments or tibial plafond depression should be treated by surgical reduction and fixation. Type IV fractures have to be treated with reduction and fixation to restore joint stability (56). The different fixation techniques are still controversially discussed, and evidence is missing regarding their influence on outcomes of the geriatric population. Direct reduction and fixation of the posterior malleolar fragment is the gold standard in particular for large, displaced, impacted or comminuted fragments. If direct reduction and fixation of the posterior malleolar fragment is chosen, a posterolateral approach is used. This approach has several advantages: the posterolateral approach enables direct visualization of the posterior malleolar fragment, provides good soft tissue coverage, the treatment of a fibular fracture can be combined over one single incision with beneficial biomechanical properties and even the medial malleolus can be reached (56, 57).

Alternatively, the fibular fracture may be approached directly through a standard lateral approach with an additional anterior minimally invasive incision in case of posterior malleolar fragment fractures and following indirect lag screw fixation (58). Percutaneous screw positioning in younger patients is performed for Bartoniček type IV fractures without an intercalary fragment or impaction (56). In the elderly patient, anterior-posterior percutaneous screw fixation of the posterior malleolar fragment is attractive because less soft tissue is exposed, supine positioning with spinal anesthesia is possible and joint congruity and stability is more important than precise anatomic articular reduction (34).

A standard lateral approach to the distal aspect of the fibula can be set slightly posterior to the fibula which provides better soft-tissue coverage to decrease the risk of exposed hardware in case of postoperative wound complications in the elderly patient (34).

Stable retention of medial malleolus fractures, which are usually treated by open and anatomically reduction, is achieved with a screw osteosynthesis or tension band wiring, favorable with penetration of both tibial cortices (59).

Trans- or supra-syndesmotic fractures of the fibula normally have to be treated with a positioning screw which can be inserted through the distal holes of the lateral fibula plate. Especially in incompliant elderly patients and patients with poor bone quality, quadricortical instead of tricortical screw insertion and two instead of one screw can be considered to have more fixation stability (60, 61). A tibia-pro-fibula technique is an option in osteoporotic multifragment fibula fractures to aim for increased resistance to torque, rotational angulation and force preventing fixation failure (62, 63, 64, 65, 66).

In osteoporotic bone, care should be taken not to overtighten syndesmotic screws as the screw threads obtain better purchase in the tibia than in the fibula. The screw head continues to advance using the plate as a washer, leading to overtightening of the tibiofibular incision or displace the fibula (67). In most cases, positioning screws can be left in place, even while increasing weight-bearing (68).

An alternative technique to stabilize the syndesmosis is the rather new suture-button augmentation. Earlier weight-bearing, better replication of the flexible tibiofibular construct and no implant removal are potential advantages over metallic screws. However, in osteoporotic bone, osteolysis near the implant might occur (69). The use of suture button fixation in patients with osteoporosis has not been analyzed yet and a more rigid fixation with screws might be favorable in patients with poor bone stock (66, 70).

## External fixation

The external fixator can be used for temporary fixation until ORIF in cases with swollen soft tissues, open fractures or dislocated fractures. The aim of a temporary external joint-bridging fixator is to restore the axis, length and torsion without causing negative effects on further therapy (30, 71).

Moreover, in persistent poor soft tissue conditions or medical peritraumatic medical complications, an external fixator can be used for definitive care in a joint bridging triangular technique (72). Alternatively, a circular external fixator can be applied if a long-lasting treatment time (>6 weeks) with an external fixator is expected in osteoporotic and diabetic ankle joints. It is a powerful tool that provides fracture stability with low soft tissue damage and permits full weight-bearing (73).

The adjuvant use of an external fixator in combination with an internal osteosynthesis with limited open or closed reduction can be considered to achieve better radiological outcomes than treatment with an external fixator alone (72).

Despite the indication of an external fixator, it is important to ensure that the fixator pins are in the correct position. These should be outside of the planned approach in order to keep the risk of contamination low. In the case of severe osteoporosis and/or inadequate stability of the pins with impossible reduction of the fracture, a retrograde thick K-wire can be inserted through the calcaneus and talus in the tibia as a temporary additional hindfoot arthrodesis. The K-wire should also penetrate the tibia cortices, to ensure later implant removal even in cases with broken K-wires. The main disadvantages of an external fixator in ankle fractures with poor bone quality are the weight and size of the construct as well as the negative effect on mobility, the need for frequent pin care pin loosening and pin-site infections (71). According to the literature, pin-site infections can be reduced by using hydroxyapatite-coated pins (74).

## Closed reduction and internal fixation, minimal invasive techniques and the fibular nail

Recently, there has been a trend toward less invasive techniques to treat ankle fractures such as distal fibular fractures. Different techniques were described ranging from minimal invasive plate osteosynthesis to closed reduction and internal fixation with nails and screws. These minimally invasive techniques result in smaller incisions with less dissection and damage to the soft tissue. Because of the limited visualization of the fracture, a full understanding of the injury mechanism, fracture geometry and proper selection of the reposition maneuvers and fixation device is necessary. Minimal invasive techniques protect the blood supply of the fragments, often enable the early function of the ankle joint and provide satisfactory clinical results when treating complex ankle fractures in patients with soft tissue problems (75, 76, 77).

Percutaneous cannulated screw fixation of the medial or lateral malleolus and intramedullary nailing for the treatment of ankle fractures in elderly or diabetic patients are effective intramedullary techniques (77, 78). In the past, intramedullary fixation techniques were not developed for the treatment of ankle fractures like they were for other fractures (e.g. tibial fractures). When using a fibular nail for ankle fractures, the literature shows less impact on the soft tissues and early postoperative mobilization with full weight-bearing (79). This results in lower postoperative complication rates and good functional results in an elderly patient population (75, 80). Most fibular nails have an anatomic design to help restoring the anatomical alignment and can be locked with two distal screws to stabilize the distal fibular fragment and two additional suprasyndesmotic positioning screws. Injuries with substantially shortened fibular fractures may require a small additional incision to help restoring the correct length and torsion. It is crucial to achieve a good entry point of the nail as otherwise it is difficult to achieve a correct restoration of the fracture. The entry point should be determined intra-operatively with the help of an image intensifier. Several studies, comparing the rate of soft tissue complications between fibular nailing and standard AO lag-screw and neutralization plate technique, report lower complication rates and higher load to failure after using the fibular nail (81, 82, 83).

## Primary retrograde nail arthrodesis

Another intramedullary nailing technique for an unstable ankle fracture in the elderly patient is the primary arthrodesis using a retrograde hindfoot nail. Especially in cases with severe soft tissue damage around the planned approach for standard implants or in low-demand geriatric patients, the use of a hindfoot nail to treat ankle fractures should be considered (64, 84). Studies show favorable outcomes regarding early rehabilitation, restoration of function and length of hospital stay (85, 86). Even in cases of non-union of the fracture, patients showed satisfying postoperative results as the nail, which can be left in place, will continue to stabilize the ankle.

## Post-traumatic and preoperative considerations

Despite treatment decision between surgical or conservative treatment, it is important to consider the individual patient during the treatment selection. The patient and the close family members should be carefully consented, and realistic outcomes have to be discussed (87). Furthermore, post-traumatic rehabilitation and a geriatric-orthopedic co-management approach to geriatric patients should be organized. Several studies have outlined the benefit of co-management between orthopedic and geriatric services in order to improve outcomes after fractures of the elderly (88, 89). It focuses on the prevention of thrombosis, treatment of comorbidities stabilization of mental status and pain control (90, 91). Optimizing medical conditions pre- and postoperatively increases the likelihood for old patients to regain former levels of activity. Comorbidities such as diabetes mellitus and osteoporosis should be diagnosed and treated to reduce readmission to hospital, complications and morbidity (92, 93). This does not only involve medication but also the nutritional status as it can be an important factor to enhance fracture healing. Malnutrition in geriatric patients often stays undetected but might lead to wound healing problems, infections and prolonged osseous consolidation. Studies showed that geriatric patients suffering from malnutrition are at a significantly higher risk for postoperative complications. Hence, the nutrition status should be analyzed prior to surgery and if malnutrition is detected it should be improved by appropriate supplementation (94).

## Postoperative care

Postoperative care, particularly in elderly patients involves soft tissue healing, early mobilization as well as osseous consolidation. Prolonged splinting or casting should be avoided as despite the lower stability, this may lead to soft-tissue compromise. When using intramedullary implants, full weight-bearing after surgical care is often possible. Otherwise, even with partial weight-bearing, immediate mobilization should be guaranteed, e.g. with the help of a walker and early physiotherapeutic care. There is no consensus among orthopedic surgeons regarding the period of non-weight-bearing after the fixation of ankle fractures (95). Until definite wound healing, regular soft tissue examinations should be performed and follow up X-rays after 6 and 12 weeks postoperatively.

## Conclusion

Ankle fractures in elderly patients are not trivial injuries and became an increasing problem. Due to different preexisting conditions like metabolic or cardiovascular diseases with poor bone quality and poor peripheral blood supply, these injuries are challenging to treat. High complication rates during conservative or surgical treatment often fail to achieve the primary status of function and mobility. Therefore, there is a high demand for safe and reliable fixation techniques like innovative intramedullary fixation with a fibular nail. The treatment of ankle fractures in elderly patients requires an individual concept. The attendant surgeon has to take the soft tissue conditions, bone quality and compliance into account. Stabil fractures and patients with absolute contraindications for operative care should be treated conservatively. Nevertheless, there is still a high rate of patients who require surgery. These patients need the best possible protection for soft tissue conditions with an adapted choice of implant. Especially in older patients with ankle fractures, there is trend toward the use of intramedullary implants.

## ICMJE conflict of interest statement

The authors have no conflicts of interest relevant to this article.

## Funding statement

The authors were not compensated for this work. The authors acknowledge support from Open Access Publishing Fund of the University of Tübingen.

## Author contribution statement

Conceptualization: PZ and CB.; methodology: PZ; validation: PZ, CK, PH, MA and CB; formal analysis: PZ; investigation: PZ, MA, CK, PH and CB; resources: CB; writing—original draft preparation: PZ, MA; writing—review and editing: PZ, CK, PH, MA and CB; visualization: PZ; supervision: CB; project administration: CB; All authors have read and agreed to the published version of the manuscript.
